# Orthogonal Polynomials with Singularly Perturbed Freud Weights

**DOI:** 10.3390/e25050829

**Published:** 2023-05-22

**Authors:** Chao Min, Liwei Wang

**Affiliations:** School of Mathematical Sciences, Huaqiao University, Quanzhou 362021, China; liweiwang@stu.hqu.edu.cn

**Keywords:** orthogonal polynomials, recurrence coefficients, singularly perturbed Freud weights, differential and difference equations

## Abstract

In this paper, we are concerned with polynomials that are orthogonal with respect to the singularly perturbed Freud weight functions. By using Chen and Ismail’s ladder operator approach, we derive the difference equations and differential-difference equations satisfied by the recurrence coefficients. We also obtain the differential-difference equations and the second-order differential equations for the orthogonal polynomials, with the coefficients all expressed in terms of the recurrence coefficients.

## 1. Introduction

Orthogonal polynomials are of great importance in Random Matrix Theory (RMT), integrable systems, numerical analysis, representation theory, etc. It is well known that classical orthogonal polynomials (Hermite, Laguerre and Jacobi) are orthogonal with respect to a weight function w(x) that satisfies the Pearson equation

(1) $$\frac{d}{dx}\left(\sigma \left(x\right)w\left(x\right)\right)=\tau \left(x\right)w\left(x\right),$$

where σ(x) is a polynomial of degree ≤2 and τ(x) is a polynomial of degree 1. Semi-classical orthogonal polynomials have a weight w(x) that satisfies the Pearson Equation (1), where σ(x) and τ(x) are polynomials with degσ(x)>2 or degτ(x)≠1 (see, e.g., ([1], Section 1.1.1)).

A motivation of this paper is the fact that the recurrence coefficients of semi-classical orthogonal polynomials are usually related to the solutions of the Painlevé equations. For example, Chen and Its [2] proved that the recurrence coefficients of orthogonal polynomials with a singularly perturbed Laguerre weight are expressed in terms of a particular Painlevé III equation. Filipuk, Van Assche and Zhang [3] showed that the recurrence coefficients of a class of semi-classical Laguerre polynomials are related to the Painlevé IV equation. Basor, Chen and Ehrhardt [4] established the relation between the recurrence coefficients of time-dependent Jacobi polynomials and the Painlevé V equation. See [5,6,7,8,9,10,11] and also the recent monograph of Van Assche [1] for more information.

A *Freud weight* is a weight function of the form ([12], Section 18.32)

w(x)=exp(−Q(x)),x∈R,

where Q(x) is real, even, nonnegative and continuously differentiable. Of special interest are the cases $Q\left(x\right)=x^{2m},\;m=1,2,3,\ldots$. In a seminal paper [9], Magnus studied the relations between the Painlevé equations and many semi-classical orthogonal polynomials, in which there are two examples of the one-parameter Freud weights:

$$w\left(x\right)=e^{-x^{4}-tx^{2}},\;\;w\left(x\right)=e^{-x^{6}-tx^{2}},\;x\in R,$$

with t∈R a parameter, and Magnus obtained a series of differential and difference equations satisfied by the recurrence coefficients of the corresponding orthogonal polynomials. See also [3,7,13,14].

In this paper, we consider the singularly perturbed Freud weight

(2) $$w\left(x;t\right):=e^{-x^{2m}-\frac{t}{x^{2}}},\;x\in R$$

with t≥0,m=1,2,3,…. When t>0, the factor $e^{-\frac{t}{x^{2}}}$ induces an infinitely strong zero at the origin. This is a semi-classical weight since it satisfies the Pearson Equation (1) with $\sigma \left(x\right)=x^{3},\;\tau \left(x\right)=-2mx^{2m+2}+3x^{2}+2t$.

Orthogonal polynomials with singularly perturbed Gaussian, Laguerre and Jacobi weights have been studied in [2,5,15]. We mention that the weights with an essential singularity at the origin, such as (2), play an important role in many mathematical and physical problems, such as the study of statistics for zeros of the Riemann zeta function [16], the calculation of finite temperature expectation values in integrable quantum field theory [17], the study of the Wigner time-delay distribution [18,19,20], etc.

Let $P_{n}\left(x;t\right),\;n=0,1,2,\ldots$ be the monic polynomials of degree *n* orthogonal with respect to the weight (2), i.e.,

(3) $$\int_{-\infty }^{\infty }P_{j}\left(x;t\right)P_{k}\left(x;t\right)w\left(x;t\right)dx=h_{j}\left(t\right)\delta_{jk},\;j,k=0,1,2,\ldots .$$

Since the weight w(x;t) is even, we have $P_{n}\left(-x;t\right)={\left(-1\right)}^{n}P_{n}\left(x;t\right)$ ([21], p. 21). Specifically, $P_{n}\left(x;t\right)$ has the expansion

(4) $$P_{n}\left(x;t\right)=x^{n}+p\left(n,t\right)x^{n-2}+\cdots ,\;n=0,1,2,\ldots ,$$

where p(n,t) is the sub-leading coefficient of $P_{n}\left(x;t\right)$, and p(0,t)=p(1,t)=0.

It is well known that the orthogonal polynomials satisfy the three-term recurrence relation ([21], pp. 18–21)

(5) $$xP_{n}\left(x;t\right)=P_{n+1}\left(x;t\right)+\beta_{n}\left(t\right)P_{n-1}\left(x;t\right),$$

with the initial conditions $P_{0}\left(x;t\right)=1,\;\beta_{0}\left(t\right)P_{-1}\left(x;t\right)=0.$ Using (3)–(5), we have two alternative expressions of $\beta_{n}\left(t\right)$:

$$\beta_{n}\left(t\right)=p\left(n,t\right)-p\left(n+1,t\right),$$

(6) $$\beta_{n}\left(t\right)=\frac{h_{n}\left(t\right)}{h_{n-1}\left(t\right)}.$$

See also [22,23] for more information about orthogonal polynomials.

From Chen and Ismail [24] (see also [25] and ([22], Chapter 3)), our orthogonal polynomials satisfy the following differential-difference equations:

(7) $$P_{n}^{\prime }\left(x\right)=-B_{n}\left(x\right)P_{n}\left(x\right)+\beta_{n}A_{n}\left(x\right)P_{n-1}\left(x\right),$$

(8) $$P_{n-1}^{\prime }\left(x\right)=\left(B_{n}\left(x\right)+v^{\prime }\left(x\right)\right)P_{n-1}\left(x\right)-A_{n-1}\left(x\right)P_{n}\left(x\right),$$

where v(x):=−lnw(x) is the potential and

(9) $$A_{n}\left(x\right):=\frac{1}{h_{n}}\int_{-\infty }^{\infty }\frac{v^{\prime }\left(x\right)-v^{\prime }\left(y\right)}{x-y}P_{n}^{2}\left(y\right)w\left(y\right)dy,$$

(10) $$B_{n}\left(x\right):=\frac{1}{h_{n-1}}\int_{-\infty }^{\infty }\frac{v^{\prime }\left(x\right)-v^{\prime }\left(y\right)}{x-y}P_{n}\left(y\right)P_{n-1}\left(y\right)w\left(y\right)dy.$$

Note that we do not display the *t*-dependence of many quantities for simplicity.

By the definitions (9) and (10) and with the aid of the three-term recurrence relation (5), it can be shown that $A_{n}\left(x\right)$ and $B_{n}\left(x\right)$ satisfy the compatibility conditions

(11) $$B_{n+1}\left(x\right)+B_{n}\left(x\right)=xA_{n}\left(x\right)-v^{\prime }\left(x\right),$$

(12) $$1+x\left(B_{n+1}\left(x\right)-B_{n}\left(x\right)\right)=\beta_{n+1}A_{n+1}\left(x\right)-\beta_{n}A_{n-1}\left(x\right).$$

Multiplying by $A_{n}\left(x\right)$ on both sides of (12) and using (11) to eliminate $xA_{n}\left(x\right)$, we have the following identity after taking a telescopic sum:

(13) $$B_{n}^{2}\left(x\right)+v^{\prime }\left(x\right)B_{n}\left(x\right)+\sum_{j=0}^{n-1}A_{j}\left(x\right)=\beta_{n}A_{n}\left(x\right)A_{n-1}\left(x\right).$$

Finally, eliminating $P_{n-1}\left(x\right)$ from (7) and (8), we obtain the second-order differential equation satisfied by the orthogonal polynomials:

(14) $$P_{n}^{\prime \prime }\left(x\right)-\left(v^{\prime }\left(x\right)+\frac{A_{n}^{\prime }\left(x\right)}{A_{n}\left(x\right)}\right)P_{n}^{\prime }\left(x\right)+\left(B_{n}^{\prime }\left(x\right)-B_{n}\left(x\right)\frac{A_{n}^{\prime }\left(x\right)}{A_{n}\left(x\right)}+\sum_{j=0}^{n-1}A_{j}\left(x\right)\right)P_{n}\left(x\right)=0,$$

where use has been made of (13) to simplify the coefficient of $P_{n}\left(x\right)$.

From the point of view of RMT, the weight (2) can be used to define a singularly perturbed Freud unitary ensemble with probability distribution

$$p\left(x_{1},x_{2},\ldots ,x_{n}\right)\prod_{k=1}^{n}dx_{k}=\frac{1}{Z_{n}}\prod_{1\leq i<j\leq n}{\left(x_{i}-x_{j}\right)}^{2}\prod_{k=1}^{n}e^{-x_{k}^{2m}-\frac{t}{x_{k}^{2}}}dx_{k},$$

where $Z_{n}$ is the partition function and can be expressed as a Hankel determinant generated by the weight (2) by using Andréief or Heine’s identity. See [26,27,28] for more information about this topic. In this respect, there are many problems to be considered, including the large *n* asymptoics of the partition function and the gap probabilities of the ensemble. We will leave these problems to a future investigation. We mention that Claeys, Krasovsky and Minakov [29] recently studied the asymptotics of the partition function and gap probabilities of a certain Freud random matrix ensemble. In addition, construction of the quadrature formulas related to the weight (2) as in [30] may be an interesting problem.

The main purpose of this paper is to derive the differential and difference equations for the recurrence coefficient $\beta_{n}$ and also the orthogonal polynomials with respect to the singularly perturbed Freud weight (2) by using Chen and Ismail’s method [24,25].

## 2. The *m* = 1 Case

In this section, we consider the simplest case (m=1) and the weight function now reads

$$w\left(x;t\right)=e^{-x^{2}-\frac{t}{x^{2}}},\;x\in R$$

with t≥0. It is also called the singularly perturbed Gaussian weight and has been studied by Min, Lyu and Chen [15] (see also [31]). One of the main results in [15] is that the authors establish the relation between the recurrence coefficient and the Painlevé III${}^{\prime }$ equation (see Theorem 3 below). The following results in Lemma 1 and Lemma 2 are obtained in [15].

**Lemma** **1.** *For this problem, we have*

(15) $$A_{n}\left(x\right)=2+\frac{R_{n}\left(t\right)}{x^{2}},$$

(16) $$B_{n}\left(x\right)=\frac{r_{n}\left(t\right)}{x}+\frac{[1-{\left(-1\right)}^{n}]t}{x^{3}},$$

*where $R_{n}\left(t\right)$ and $r_{n}\left(t\right)$ are the auxiliary quantities defined by*

$$R_{n}\left(t\right):=\frac{2t}{h_{n}}\int_{-\infty }^{\infty }\frac{1}{y^{2}}P_{n}^{2}\left(y\right)w\left(y\right)dy,$$

$$r_{n}\left(t\right):=\frac{2t}{h_{n-1}}\int_{-\infty }^{\infty }\frac{1}{y^{3}}P_{n}\left(y\right)P_{n-1}\left(y\right)w\left(y\right)dy.$$

Substituting (15) and (16) into (11) and (13), we have the following.

**Lemma** **2.** *The auxiliary quantities $R_{n}\left(t\right),r_{n}\left(t\right)$ and the recurrence coefficient $\beta_{n}$ satisfy the following relations:*

(17) $$R_{n}\left(t\right)=r_{n+1}\left(t\right)+r_{n}\left(t\right),$$

(18) $$\beta_{n}=\frac{n+r_{n}\left(t\right)}{2},$$

(19) $$-2{\left(-1\right)}^{n}t\;r_{n}\left(t\right)=\beta_{n}R_{n}\left(t\right)R_{n-1}\left(t\right),$$

(20) $$r_{n}^{2}\left(t\right)+2\left[1-{\left(-1\right)}^{n}\right]t+\sum_{j=0}^{n-1}R_{j}\left(t\right)=2\beta_{n}R_{n-1}\left(t\right)+2\beta_{n}R_{n}\left(t\right).$$

**Theorem** **1.** *The recurrence coefficient $\beta_{n}$ satisfies the nonlinear* ***second**-order difference equation*

$$\beta_{n}\left(2\beta_{n+1}+2\beta_{n}-2n-1\right)\left(2\beta_{n}+2\beta_{n-1}-2n+1\right)+2{\left(-1\right)}^{n}t\left(2\beta_{n}-n\right)=0.$$

**Proof.**  From (18) and (17), we have

(21) $$r_{n}\left(t\right)=2\beta_{n}-n,$$

(22) $$R_{n}\left(t\right)=2\beta_{n+1}+2\beta_{n}-2n-1.$$

Substituting (21) and (22) into (19), we establish the theorem. □

**Theorem** **2.** *The recurrence coefficient $\beta_{n}$ satisfies the differential-difference equation*

$$t\beta_{n}^{\prime }\left(t\right)=\beta_{n}\left(\beta_{n-1}-\beta_{n+1}+1\right).$$

**Proof.**  Taking a derivative with respect to *t* in the orthogonality condition

$$h_{n}\left(t\right)=\int_{-\infty }^{\infty }P_{n}^{2}\left(x,t\right)e^{-x^{2}-\frac{t}{x^{2}}}dx,\;n=0,1,2,\ldots ,$$

we find

$$2t\frac{d}{dt}\ln h_{n}\left(t\right)=-R_{n}\left(t\right).$$

Using (6), we have

$$2t\beta_{n}^{\prime }\left(t\right)=\beta_{n}\left(R_{n-1}\left(t\right)-R_{n}\left(t\right)\right).$$

Substituting (22) into the above, we obtain the desired result. □

**Theorem** **3.** *The auxiliary quantity $R_{n}\left(t\right)$, related to the recurrence coefficient $\beta_{n}$ by*

$$R_{n}\left(t\right)=2\beta_{n+1}+2\beta_{n}-2n-1,$$

*satisfies a particular Painlevé III${}^{\prime }$ equation ([32], (2)):*

$$R_{n}^{\prime \prime }\left(t\right)=\frac{{\left(R_{n}^{\prime }\left(t\right)\right)}^{2}}{R_{n}\left(t\right)}-\frac{R_{n}^{\prime }\left(t\right)}{t}+\frac{\left(2n+1\right)R_{n}^{2}\left(t\right)}{4t^{2}}-\frac{{\left(-1\right)}^{n}}{t}+\frac{R_{n}^{3}\left(t\right)}{4t^{2}}-\frac{4}{R_{n}\left(t\right)}.$$

**Proof.**  This is obtained by eliminating $r_{n}\left(t\right)$ from the coupled Riccati equations satisfied by $R_{n}\left(t\right)$ and $r_{n}\left(t\right)$. See [15] for details. □

**Theorem** **4.** *The orthogonal polynomials $P_{n}\left(x\right)$ satisfy the differential-difference Equations (7) and (8), and the second-order differential Equation (14) with*

(23) $$A_{n}\left(x\right)=2+\frac{2\beta_{n+1}+2\beta_{n}-2n-1}{x^{2}},\;B_{n}\left(x\right)=\frac{2\beta_{n}-n}{x}+\frac{[1-{\left(-1\right)}^{n}]t}{x^{3}},$$

(24) $$\sum_{j=0}^{n-1}A_{j}\left(x\right)=2n+\frac{4\beta_{n}\left(\beta_{n+1}+\beta_{n}+\beta_{n-1}-n\right)-n^{2}-2\left[1-{\left(-1\right)}^{n}\right]t}{x^{2}},$$

*and $v^{\prime }\left(x\right)=2x-\frac{2t}{x^{3}}$.*

**Proof.**  Substituting (22) and (21) into (15) and (16), we have the expressions of $A_{n}\left(x\right)$ and $B_{n}\left(x\right)$ in (23). From (15) and (20), we find

$$\sum_{j=0}^{n-1}A_{j}\left(x\right)=2n+\frac{\sum_{j=0}^{n-1}R_{j}\left(t\right)}{x^{2}}=2n+\frac{2\beta_{n}\left(R_{n-1}\left(t\right)+R_{n}\left(t\right)\right)-r_{n}^{2}\left(t\right)-2\left[1-{\left(-1\right)}^{n}\right]t}{x^{2}}.$$

Substituting (22) and (21) into the above, we obtain (24). □

## 3. The *m* = 2 Case

In this section, we consider the m=2 case and the weight function is

$$w\left(x;t\right)=e^{-x^{4}-\frac{t}{x^{2}}},\;x\in R$$

with t≥0. It is easy to see that the potential is

$$v\left(x\right)=-\ln w\left(x\right)=x^{4}+\frac{t}{x^{2}}.$$

It follows that

$$v^{\prime }\left(x\right)=4x^{3}-\frac{2t}{x^{3}},$$

and

(25) $$\frac{v^{\prime }\left(x\right)-v^{\prime }\left(y\right)}{x-y}=4x^{2}+4xy+4y^{2}+\frac{2t}{xy^{3}}+\frac{2t}{x^{2}y^{2}}+\frac{2t}{x^{3}y}.$$

**Lemma** **3.** *We have*

(26) $$A_{n}\left(x\right)=4x^{2}+4\left(\beta_{n}+\beta_{n+1}\right)+\frac{R_{n}\left(t\right)}{x^{2}},$$

(27) $$B_{n}\left(x\right)=4x\beta_{n}+\frac{r_{n}\left(t\right)}{x}+\frac{\left[1-{\left(-1\right)}^{n}\right]t}{x^{3}},$$

*where $R_{n}\left(t\right)$ and $r_{n}\left(t\right)$ are the auxiliary quantities given by*

$$R_{n}\left(t\right):=\frac{2t}{h_{n}}\int_{-\infty }^{\infty }\frac{1}{y^{2}}P_{n}^{2}\left(y\right)w\left(y\right)dy,$$

$$r_{n}\left(t\right):=\frac{2t}{h_{n-1}}\int_{-\infty }^{\infty }\frac{1}{y^{3}}P_{n}\left(y\right)P_{n-1}\left(y\right)w\left(y\right)dy.$$

**Proof.**  Substituting (25) into the definition of $A_{n}\left(x\right)$ in (9) and using the parity of the integrand, we obtain

(28) $$A_{n}\left(x\right)=4x^{2}+\frac{4}{h_{n}}\int_{-\infty }^{\infty }y^{2}P_{n}^{2}\left(y\right)w\left(y\right)dy+\frac{2t}{x^{2}h_{n}}\int_{-\infty }^{\infty }\frac{1}{y^{2}}P_{n}^{2}\left(y\right)w\left(y\right)dy.$$

By the three-term recurrence relation (5), we have

$$y^{2}P_{n}^{2}\left(y\right)={\left(P_{n+1}\left(y\right)+\beta_{n}P_{n-1}\left(y\right)\right)}^{2}=P_{n+1}^{2}\left(y\right)+\beta_{n}^{2}P_{n-1}^{2}\left(y\right)+2\beta_{n}P_{n+1}\left(y\right)P_{n-1}\left(y\right).$$

It follows that

(29) $$\frac{1}{h_{n}}\int_{-\infty }^{\infty }y^{2}P_{n}^{2}\left(y\right)w\left(y\right)dy=\beta_{n}+\beta_{n+1},$$

where use has been made of (6). Inserting (29) into (28) gives (26).

Similarly, substituting (25) into the definition of $B_{n}\left(x\right)$ in (10), we have

$$B_{n}\left(x\right)=4x\beta_{n}+\frac{2t}{xh_{n-1}}\int_{-\infty }^{\infty }\frac{1}{y^{3}}P_{n}\left(y\right)P_{n-1}\left(y\right)w\left(y\right)dy+\frac{2t}{x^{3}h_{n-1}}\int_{-\infty }^{\infty }\frac{1}{y}P_{n}\left(y\right)P_{n-1}\left(y\right)w\left(y\right)dy.$$

It is easy to see that

(30) $$\begin{array}{ccc} \frac{1}{h_{n-1}}\int_{-\infty }^{\infty }\frac{1}{y}P_{n}\left(y\right)P_{n-1}\left(y\right)w\left(y\right)dy & = & \left\{\begin{array}{cc} & 0,\;n=0,2,4,\ldots ,\\ & 1,\;n=1,3,5,\ldots \end{array}\right.\\ & = & \frac{1-{\left(-1\right)}^{n}}{2}. \end{array}$$

Then, we arrive at (27). The proof is complete. □

**Theorem** **5.** *The recurrence coefficient $\beta_{n}$ satisfies the nonlinear* ***fourth**-order difference equation*

(31) $$\begin{array}{c} \beta_{n}\left[4\beta_{n-2}\beta_{n-1}+4{\left(\beta_{n-1}+\beta_{n}\right)}^{2}+4\beta_{n}\beta_{n+1}-2n+1\right]\left[4\beta_{n-1}\beta_{n}+4{\left(\beta_{n}+\beta_{n+1}\right)}^{2}+4\beta_{n+1}\beta_{n+2}-2n-1\right]\\ +2{\left(-1\right)}^{n}t\left[4\beta_{n}\left(\beta_{n-1}+\beta_{n}+\beta_{n+1}\right)-n\right]=0. \end{array}$$

**Proof.**  Substituting (26) and (27) into (11), we find

(32) $$R_{n}\left(t\right)=r_{n}\left(t\right)+r_{n+1}\left(t\right).$$

Similarly, substituting (26) and (27) into (13), we obtain the following four identities:

(33) $$n+r_{n}\left(t\right)-4\beta_{n}\left(\beta_{n-1}+\beta_{n}+\beta_{n+1}\right)=0,$$

(34) $$\beta_{n}R_{n-1}\left(t\right)R_{n}\left(t\right)+2{\left(-1\right)}^{n}tr_{n}\left(t\right)=0,$$

(35) $$r_{n}^{2}\left(t\right)-8{\left(-1\right)}^{n}t\beta_{n}-4\beta_{n}\left(\beta_{n-1}+\beta_{n}\right)R_{n}\left(t\right)-4\beta_{n}\left(\beta_{n}+\beta_{n+1}\right)R_{n-1}\left(t\right)+\sum_{j=0}^{n-1}R_{j}\left(t\right)=0,$$

(36) $$\left[1-{\left(-1\right)}^{n}\right]t+2\beta_{n}r_{n}\left(t\right)-\beta_{n}\left(R_{n-1}\left(t\right)+R_{n}\left(t\right)\right)-4\beta_{n}\left(\beta_{n-1}+\beta_{n}\right)\left(\beta_{n}+\beta_{n+1}\right)+\sum_{j=0}^{n-1}\left(\beta_{j}+\beta_{j+1}\right)=0.$$

From (33) and (32), we can express $r_{n}\left(t\right)$ and $R_{n}\left(t\right)$ in terms of the recurrence coefficient $\beta_{n}$:

(37) $$r_{n}\left(t\right)=4\beta_{n}\left(\beta_{n-1}+\beta_{n}+\beta_{n+1}\right)-n,$$

(38) $$R_{n}\left(t\right)=4\beta_{n-1}\beta_{n}+4{\left(\beta_{n}+\beta_{n+1}\right)}^{2}+4\beta_{n+1}\beta_{n+2}-2n-1.$$

Substituting (37) and (38) into (34), we obtain (31). □

**Theorem** **6.** *The recurrence coefficient $\beta_{n}$ satisfies the differential-difference equation*

$$t\beta_{n}^{\prime }\left(t\right)=\beta_{n}\left[1+2\beta_{n-1}\left(\beta_{n-2}+\beta_{n-1}+\beta_{n}\right)-2\beta_{n+1}\left(\beta_{n}+\beta_{n+1}+\beta_{n+2}\right)\right].$$

**Proof.**  Taking a derivative with respect to *t* in the equality

$$h_{n}\left(t\right)=\int_{-\infty }^{\infty }P_{n}^{2}\left(x;t\right)e^{-x^{4}-\frac{t}{x^{2}}}dx,$$

we find

$$2t\frac{d}{dt}\ln h_{n}\left(t\right)=-R_{n}\left(t\right)$$

and then

$$2t\beta_{n}^{\prime }\left(t\right)=\beta_{n}\left(R_{n-1}\left(t\right)-R_{n}\left(t\right)\right).$$

Substituting (38) into the above gives the desired result. □

**Theorem** **7.** *The orthogonal polynomials $P_{n}\left(x\right)$ satisfy the differential-difference Equations (7) and (8), and the second-order differential Equation (14) with*

(39) $$A_{n}\left(x\right)=4x^{2}+4\left(\beta_{n}+\beta_{n+1}\right)+\frac{4\beta_{n-1}\beta_{n}+4{\left(\beta_{n}+\beta_{n+1}\right)}^{2}+4\beta_{n+1}\beta_{n+2}-2n-1}{x^{2}},$$

(40) $$B_{n}\left(x\right)=4x\beta_{n}+\frac{4\beta_{n}\left(\beta_{n-1}+\beta_{n}+\beta_{n+1}\right)-n}{x}+\frac{[1-{\left(-1\right)}^{n}]t}{x^{3}},$$

(41) $$\begin{array}{ccc} \sum_{j=0}^{n-1}A_{j}\left(x\right) & = & 4nx^{2}+16\beta_{n}\left[\beta_{n-1}\left(\beta_{n-2}+\beta_{n-1}+2\beta_{n}+\beta_{n+1}\right)+{\left(\beta_{n}+\beta_{n+1}\right)}^{2}+\beta_{n+1}\beta_{n+2}\right]\\ & & -8n\beta_{n}-4\left[1-{\left(-1\right)}^{n}\right]t-\frac{{\left[4\beta_{n}\left(\beta_{n-1}+\beta_{n}+\beta_{n+1}\right)-n\right]}^{2}-8{\left(-1\right)}^{n}t\beta_{n}}{x^{2}}\\ & & +\frac{4\beta_{n}\left(\beta_{n-1}+\beta_{n}\right)\left[4\beta_{n-1}\beta_{n}+4{\left(\beta_{n}+\beta_{n+1}\right)}^{2}+4\beta_{n+1}\beta_{n+2}-2n-1\right]}{x^{2}}\\ & & +\frac{4\beta_{n}\left(\beta_{n}+\beta_{n+1}\right)\left[4\beta_{n-2}\beta_{n-1}+4{\left(\beta_{n-1}+\beta_{n}\right)}^{2}+4\beta_{n}\beta_{n+1}-2n+1\right]}{x^{2}},\\ & & \mathrm{and}v^{\prime }\left(x\right)=4x^{3}-\frac{2t}{x^{3}}. \end{array}$$

**Proof.**  Substituting (38) and (37) into (26) and (27), we obtain (39) and (40), respectively. From (26), we have

(42) $$\sum_{j=0}^{n-1}A_{j}\left(x\right)=4nx^{2}+4\sum_{j=0}^{n-1}\left(\beta_{j}+\beta_{j+1}\right)+\frac{\sum_{j=0}^{n-1}R_{j}\left(t\right)}{x^{2}}.$$

Using (36) and (35), we find

(43) $$\sum_{j=0}^{n-1}\left(\beta_{j}+\beta_{j+1}\right)=-\left[1-{\left(-1\right)}^{n}\right]t-2\beta_{n}r_{n}\left(t\right)+\beta_{n}\left(R_{n-1}\left(t\right)+R_{n}\left(t\right)\right)+4\beta_{n}\left(\beta_{n-1}+\beta_{n}\right)\left(\beta_{n}+\beta_{n+1}\right),$$

(44) $$\sum_{j=0}^{n-1}R_{j}\left(t\right)=-r_{n}^{2}\left(t\right)+8{\left(-1\right)}^{n}t\beta_{n}+4\beta_{n}\left(\beta_{n-1}+\beta_{n}\right)R_{n}\left(t\right)+4\beta_{n}\left(\beta_{n}+\beta_{n+1}\right)R_{n-1}\left(t\right).$$

Inserting (43) and (44) into (42), and eliminating $R_{n}\left(t\right),R_{n-1}\left(t\right)$ and $r_{n}\left(t\right)$ by (38) and (37), we obtain (41). This completes the proof. □

In the m=2 case, we find that the expressions of $A_{n}\left(x\right)$ and $B_{n}\left(x\right)$ include the terms of the recurrence coefficient $\beta_{n}$, which is different from the m=1 case. This leads to the result that $\beta_{n}$ and $r_{n}\left(t\right)$ do not have a simple relation as in the m=1 case by using the compatibility conditions. Thus, we cannot derive the coupled Riccati equations for the auxiliary quantities $R_{n}\left(t\right)$ and $r_{n}\left(t\right)$, and the second-order differential equations for $R_{n}\left(t\right)$ and $r_{n}\left(t\right)$. Therefore, the relation between our problem and the Painlevé equations is not clear in the m=2 case. A similar phenomenon arises in the next m=3 case; see also [33].

## 4. The *m* = 3 Case

In this section, we consider the weight function

(45) $$w\left(x;t\right)=e^{-x^{6}-\frac{t}{x^{2}}},\;x\in R$$

with t≥0. We have

$$v\left(x\right)=-\ln w\left(x\right)=x^{6}+\frac{t}{x^{2}}.$$

It follows that

$$v^{\prime }\left(x\right)=6x^{5}-\frac{2t}{x^{3}},$$

and

(46) $$\frac{v^{\prime }\left(x\right)-v^{\prime }\left(y\right)}{x-y}=6x^{4}+6x^{3}y+6x^{2}y^{2}+6xy^{3}+6y^{4}+\frac{2t}{xy^{3}}+\frac{2t}{x^{2}y^{2}}+\frac{2t}{x^{3}y}.$$

**Lemma** **4.** *We have*

(47) $$A_{n}\left(x\right)=6x^{4}+6x^{2}\left(\beta_{n}+\beta_{n+1}\right)+6R_{n}^{*}\left(t\right)+\frac{R_{n}\left(t\right)}{x^{2}},$$

(48) $$B_{n}\left(x\right)=6x^{3}\beta_{n}+6xr_{n}^{*}\left(t\right)+\frac{r_{n}\left(t\right)}{x}+\frac{[1-{\left(-1\right)}^{n}]t}{x^{3}},$$

where $R_{n}^{*}\left(t\right)$, $R_{n}\left(t\right)$, $r_{n}^{*}\left(t\right)$ and $r_{n}\left(t\right)$ are the auxiliary quantities given by

$$R_{n}^{*}\left(t\right)=\frac{1}{h_{n}}\int_{-\infty }^{\infty }y^{4}P_{n}^{2}\left(y\right)w\left(y\right)dy,$$

$$R_{n}\left(t\right)=\frac{2t}{h_{n}}\int_{-\infty }^{\infty }\frac{1}{y^{2}}P_{n}^{2}\left(y\right)w\left(y\right)dy,$$

$$r_{n}^{*}\left(t\right)=\frac{1}{h_{n-1}}\int_{-\infty }^{\infty }y^{3}P_{n}\left(y\right)P_{n-1}\left(y\right)w\left(y\right)dy,$$

$$r_{n}\left(t\right)=\frac{2t}{h_{n-1}}\int_{-\infty }^{\infty }\frac{1}{y^{3}}P_{n}\left(y\right)P_{n-1}\left(y\right)w\left(y\right)dy.$$

**Proof.**  Substituting (46) into (9) and (10) and noting that formulas (29) and (30) still hold for the weight (45), we obtain the desired results. □

**Theorem** **8.** *The recurrence coefficient $\beta_{n}$ satisfies the nonlinear* ***sixth**-order difference equation*

$$\begin{array}{c} \beta_{n}[6\beta_{n-2}\beta_{n-1}\left(\beta_{n-3}+\beta_{n-2}+2\beta_{n-1}+2\beta_{n}\right)+6\beta_{n}\beta_{n+1}\left(2\beta_{n-1}+2\beta_{n}+\beta_{n+1}+\beta_{n+2}\right)\\ +18\beta_{n-1}\beta_{n}\left(\beta_{n-1}+\beta_{n}\right)+6\beta_{n-1}^{3}+6\beta_{n}^{3}-2n+1][6\beta_{n-1}\beta_{n}\left(\beta_{n-2}+\beta_{n-1}+2\beta_{n}+2\beta_{n+1}\right)\\ +6\beta_{n+1}\beta_{n+2}\left(2\beta_{n}+2\beta_{n+1}+\beta_{n+2}+\beta_{n+3}\right)+18\beta_{n}\beta_{n+1}\left(\beta_{n}+\beta_{n+1}\right)+6\beta_{n}^{3}+6\beta_{n+1}^{3}-2n-1]\\ +2{\left(-1\right)}^{n}t\left[6\beta_{n-1}\beta_{n}\left(\beta_{n-2}+\beta_{n-1}+2\beta_{n}+\beta_{n+1}\right)+6\beta_{n}{\left(\beta_{n}+\beta_{n+1}\right)}^{2}+6\beta_{n}\beta_{n+1}\beta_{n+2}-n\right]=0. \end{array}$$

**Proof.**  Substituting (47) and (48) into (11), we find

(49) $$R_{n}^{*}=r_{n}^{*}+r_{n+1}^{*},$$

(50) $$R_{n}=r_{n}+r_{n+1}.$$

Similarly, substituting (47) and (48) into (13), we obtain the following six identities:

(51) $$r_{n}^{*}-\beta_{n}\left(\beta_{n-1}+\beta_{n}+\beta_{n+1}\right)=0,$$

(52) $$n+r_{n}+12\beta_{n}r_{n}^{*}-6\beta_{n}\left(R_{n-1}^{*}+R_{n}^{*}\right)-6\beta_{n}\left(\beta_{n-1}+\beta_{n}\right)\left(\beta_{n}+\beta_{n+1}\right)=0,$$

(53) $$\beta_{n}R_{n-1}R_{n}+2{\left(-1\right)}^{n}tr_{n}=0,$$

(54) $$r_{n}^{2}-12{\left(-1\right)}^{n}tr_{n}^{*}-6\beta_{n}\left(R_{n-1}R_{n}^{*}+R_{n-1}^{*}R_{n}\right)+\sum_{j=0}^{n-1}R_{j}=0,$$

(55) $$2r_{n}r_{n}^{*}-2{\left(-1\right)}^{n}t\beta_{n}-6\beta_{n}R_{n-1}^{*}R_{n}^{*}-\left(\beta_{n-1}+\beta_{n}\right)\beta_{n}R_{n}-\left(\beta_{n}+\beta_{n+1}\right)\beta_{n}R_{n-1}+\sum_{j=0}^{n-1}R_{j}^{*}=0,$$

(56) $$\left[1-{\left(-1\right)}^{n}\right]t+6{\left(r_{n}^{*}\right)}^{2}+2\beta_{n}r_{n}-6\left(\beta_{n-1}+\beta_{n}\right)\beta_{n}R_{n}^{*}-6\left(\beta_{n}+\beta_{n+1}\right)\beta_{n}R_{n-1}^{*}-\beta_{n}\left(R_{n-1}+R_{n}\right)+\sum_{j=0}^{n-1}\left(\beta_{j}+\beta_{j+1}\right)=0.$$

From (51) and (49), we can express $r_{n}^{*}$ and $R_{n}^{*}$ in terms of the recurrence coefficient,

(57) $$r_{n}^{*}=\beta_{n}\left(\beta_{n-1}+\beta_{n}+\beta_{n+1}\right),$$

(58) $$R_{n}^{*}=\beta_{n-1}\beta_{n}+{\left(\beta_{n}+\beta_{n+1}\right)}^{2}+\beta_{n+1}\beta_{n+2}.$$

It follows from (52) and (50) that $r_{n}$ and $R_{n}$ can also be expressed in terms of $\beta_{n}$,

(59) $$r_{n}=6\beta_{n}\left[\beta_{n-1}\left(\beta_{n-2}+\beta_{n-1}+2\beta_{n}+\beta_{n+1}\right)+{\left(\beta_{n}+\beta_{n+1}\right)}^{2}+\beta_{n+1}\beta_{n+2}\right]-n,$$

(60) $$\begin{array}{ccc} R_{n} & = & 6\beta_{n-1}\beta_{n}\left(\beta_{n-2}+\beta_{n-1}+2\beta_{n}+2\beta_{n+1}\right)+6\beta_{n+1}\beta_{n+2}\left(2\beta_{n}+2\beta_{n+1}+\beta_{n+2}+\beta_{n+3}\right)\\ & & +18\beta_{n}\beta_{n+1}\left(\beta_{n}+\beta_{n+1}\right)+6\beta_{n}^{3}+6\beta_{n+1}^{3}-2n-1. \end{array}$$

Substituting (59) and (60) into (53), we establish the theorem. □

**Remark** **1.** The expressions of $r_{n}^{*}$ and $R_{n}^{*}$ in (57) and (58) can also be obtained by using the three-term recurrence relation and the orthogonality from their definitions in Lemma 4.

**Theorem** **9.** *The recurrence coefficient $\beta_{n}\left(t\right)$ satisfies the differential-difference equation*

$$\begin{array}{ccc} t\beta_{n}^{\prime }\left(t\right) & = & \beta_{n}[3\beta_{n-2}\beta_{n-1}\left(\beta_{n-3}+\beta_{n-2}+2\beta_{n-1}+\beta_{n}\right)+3\beta_{n-1}{\left(\beta_{n-1}+\beta_{n}\right)}^{2}-3\beta_{n+1}{\left(\beta_{n}+\beta_{n+1}\right)}^{2}\\ & & -3\beta_{n+1}\beta_{n+2}\left(\beta_{n}+2\beta_{n+1}+\beta_{n+2}+\beta_{n+3}\right)+1]. \end{array}$$

**Proof.**  Taking a derivative with respect to *t* in the equality

$$h_{n}\left(t\right)=\int_{-\infty }^{\infty }P_{n}^{2}\left(x;t\right)e^{-x^{6}-\frac{t}{x^{2}}}dx$$

gives

$$2t\frac{d}{dt}\ln h_{n}\left(t\right)=-R_{n}\left(t\right)$$

and then

$$2t\beta_{n}^{\prime }\left(t\right)=\beta_{n}\left(R_{n-1}\left(t\right)-R_{n}\left(t\right)\right).$$

Substituting (60) into the above gives the desired result. □

**Theorem** **10.** *The orthogonal polynomials $P_{n}\left(x\right)$ satisfy the differential-difference Equations (7) and (8), and the second-order differential Equation (14) with*

(61) $$\begin{array}{ccc} A_{n}\left(x\right) & = & 6x^{4}+6x^{2}\left(\beta_{n}+\beta_{n+1}\right)+6\left[\beta_{n-1}\beta_{n}+{\left(\beta_{n}+\beta_{n+1}\right)}^{2}+\beta_{n+1}\beta_{n+2}\right]\\ & & +\frac{6\beta_{n-1}\beta_{n}\left(\beta_{n-2}+\beta_{n-1}+2\beta_{n}+2\beta_{n+1}\right)+6\beta_{n+1}\beta_{n+2}\left(2\beta_{n}+2\beta_{n+1}+\beta_{n+2}+\beta_{n+3}\right)}{x^{2}}\\ & & +\frac{18\beta_{n}\beta_{n+1}\left(\beta_{n}+\beta_{n+1}\right)+6\beta_{n}^{3}+6\beta_{n+1}^{3}-2n-1}{x^{2}}, \end{array}$$

(62) $$\begin{array}{ccc} B_{n}\left(x\right) & = & 6x^{3}\beta_{n}+6x\beta_{n}\left(\beta_{n-1}+\beta_{n}+\beta_{n+1}\right)+\frac{[1-{\left(-1\right)}^{n}]t}{x^{3}}\\ & & +\frac{6\beta_{n}\left[\beta_{n-1}\left(\beta_{n-2}+\beta_{n-1}+2\beta_{n}+\beta_{n+1}\right)+{\left(\beta_{n}+\beta_{n+1}\right)}^{2}+\beta_{n+1}\beta_{n+2}\right]-n}{x}, \end{array}$$

(63) $$\begin{array}{ccc} \sum_{j=0}^{n-1}A_{j}\left(x\right) & = & 6nx^{4}-6x^{2}[\left(1-{\left(-1\right)}^{n}\right)t+6{\left(r_{n}^{*}\right)}^{2}+2\beta_{n}r_{n}-6\left(\beta_{n-1}+\beta_{n}\right)\beta_{n}R_{n}^{*}-6\left(\beta_{n}+\beta_{n+1}\right)\beta_{n}R_{n-1}^{*}\\ & & -\beta_{n}\left(R_{n-1}+R_{n}\right)]-6[2r_{n}r_{n}^{*}-2{\left(-1\right)}^{n}t\beta_{n}-6\beta_{n}R_{n-1}^{*}R_{n}^{*}-\left(\beta_{n-1}+\beta_{n}\right)\beta_{n}R_{n}\\ & & -\left(\beta_{n}+\beta_{n+1}\right)\beta_{n}R_{n-1}]-\frac{r_{n}^{2}-12{\left(-1\right)}^{n}tr_{n}^{*}-6\beta_{n}\left(R_{n-1}R_{n}^{*}+R_{n-1}^{*}R_{n}\right)}{x^{2}}, \end{array}$$

where $r_{n}^{*},R_{n}^{*},r_{n}$ and $R_{n}$ are given by (57), (58), (59) and (60), and $v^{\prime }\left(x\right)=6x^{5}-\frac{2t}{x^{3}}$.

**Proof.**  Inserting (58) and (60) into (47) and inserting (57) and (59) into (48), we obtain (61) and (62), respectively. From (47), we have

$$\sum_{j=0}^{n-1}A_{j}\left(x\right)=6nx^{4}+6x^{2}\sum_{j=0}^{n-1}\left(\beta_{j}+\beta_{j+1}\right)+6\sum_{j=0}^{n-1}R_{j}^{*}+\frac{\sum_{j=0}^{n-1}R_{j}}{x^{2}}.$$

Eliminating $\sum_{j=0}^{n-1}\left(\beta_{j}+\beta_{j+1}\right),\sum_{j=0}^{n-1}R_{j}^{*}$ and $\sum_{j=0}^{n-1}R_{j}$ by using (56), (55) and (54), we obtain (63). □

**Remark** **2.** *From (63), we see that $\sum_{j=0}^{n-1}A_{j}\left(x\right)$ can be expressed in terms of the recurrence coefficients $\beta_{n-3},\beta_{n-2},\beta_{n-1},\beta_{n},\beta_{n+1},\beta_{n+2},\beta_{n+3}$. We do not write the explicit expression since it is very long.*

## 5. Conclusions

In this paper, we study the singularly perturbed Freud weights (2), the associated orthogonal polynomials and the recurrence coefficients in the m=1,2,3 cases. We derive the differential and difference equations for the recurrence coefficients and the orthogonal polynomials in the three cases. It can be seen that for increasing *m*, the order of the difference equations increases by 2 in each case. We can use our method to consider the higher-order singularly perturbed Freud weights. However, one can imagine that the results for the differential and difference equations will be more and more complicated. Finally, we find that the recurrence coefficient is related to the Painlevé III equation when m=1, but it is not clear whether there is any connection to the Painlevé equations when m≥2.
